# Resveratrol intake during pregnancy and lactation re-programs adiposity and ameliorates leptin resistance in male progeny induced by maternal high-fat/high sucrose plus postnatal high-fat/high sucrose diets via fat metabolism regulation

**DOI:** 10.1186/s12944-020-01349-w

**Published:** 2020-07-25

**Authors:** Ta-Yu Liu, Hong-Ren Yu, Ching-Chou Tsai, Li-Tung Huang, Chih-Cheng Chen, Jium-Ming Sheen, Mao-Meng Tiao, You-Lin Tain, I-Chun Lin, Yun-Ju Lai, Yu-Ju Lin, Te-Yao Hsu

**Affiliations:** 1 Department of Pediatrics, Kaohsiung Chang Gung Memorial Hospital, Graduate Institute of Clinical Medical Science, Chang Gung University College of Medicine, Kaohsiung, Taiwan; 2 Department of Obstetrics and Gynecology, Kaohsiung Chang Gung Memorial Hospital, #123, Ta-Pei Road, Niao-Sung District, Kaohsiung, Taiwan

**Keywords:** Maternal resveratrol, Reprograms, Prenatal, Postnatal, High-fat diet, Adiposity, Leptin

## Abstract

**Background:**

Maternal obesity is an emerging problem in the modern world. Growing evidence suggests that intrauterine high-fat (HF) exposure may predispose progeny to subsequent metabolic challenges. Progeny born to mothers who ate an HF diet also tends to eat an HF diet when growing and aggravate metabolic issues. Thus, the generational transmission of obesity is cyclical. Developing a strategy to prevent the occurrence of metabolic syndrome related to prenatal and/or postnatal HF diet is important. In this study, the reprogramming effects of maternal resveratrol treatment for the progeny with maternal HF/postnatal HF diets were investigated.

**Methods:**

Sprague-Dawley dams were fed either a control or a high-fat/high sucrose diet (HFHS) from mating to lactation. After weaning, the progeny was fed chow or an HF diet. Four experimental groups were yielded: CC (maternal/postnatal control diet), HC (maternal HF/postnatal control diet), CH (maternal control/postnatal HFHS diet), and HH (maternal/postnatal HFHS diet). A fifth group (HRH) received a maternal HFHS diet plus maternal resveratrol treatment and a postnatal chow diet to study the effects of maternal resveratrol therapy.

**Results:**

Maternal resveratrol treatment lessened the weight and adiposity of progeny that were programmed by combined prenatal and postnatal HFHS diets. Maternal resveratrol therapy ameliorated the decreased abundance of the sirtuin 1 (SIRT1) enzyme in retroperitoneal tissue and the altered leptin/soluble leptin receptor ratio of progeny. Maternal resveratrol therapy also decreased lipogenesis and increased lipolysis for progeny.

**Conclusions:**

Maternal resveratrol intervention can prevent adiposity programmed by maternal and postnatal HFHS diets by inducing lipid metabolic modulation. This study offers a novel reprogramming role for the effect of maternal resveratrol supplements against obesity.

## Background

Obesity is one of the most important health challenges in the modern world. Many epidemiological studies have demonstrated a rapid increase in the prevalence of obesity [1]. Obesity is commonly associated with adverse consequences including chronic inflammation, type 2 diabetes, glucose intolerance, hypertension, cardiovascular problems, chronic kidney disease, and non-alcoholic fatty liver disease [2–8]. The equilibrium between energy intake (food consumption) and energy expenditure (basal metabolism, physical activity, and thermogenesis) is closely regulated in individuals. Obesity takes place when energy intake exceeds energy expenditure and is characterized by an excess of adipose tissue. Adipose tissue is now considered an active metabolic tissue that regulates metabolism and inflammation through adipokines, such as leptin, visfatin, apelin, resistin, and adiponectin [7, 9–12]. Leptin, the first adipocytokine recognized, acts on the brain and promotes satiety and energy consumption. Apart from the central nervous system, leptin receptors are expressed in many peripheral tissues and promote inflammation, glomerular endothelial cell proliferation, lipid metabolism, and insulin resistance [11, 13–15]. Despite adequate leptin regulation, however, leptin resistance can occur in association with enhanced circulating leptin levels in obese humans resulting from relative insensitivity to leptin at its site of action [16].

The developmental origins of health and disease (DOHaD) is a widely accepted concept today [17]. In determining the development of human diseases in adulthood, DOHaD emphasizes the role of prenatal and perinatal exposure to environmental factors [18–21]. Many pieces of evidence strengthening DOHaD science have been observed from animal models and human studies. Maternal obesity is an emerging problem globally, with over 30% of women of child-bearing age being classified as obese [22]. Growing evidence suggests that an intrauterine high-fat (HF) environment may predispose progeny to subsequent metabolic challenges, such as hypertension, diabetes, and metabolic syndrome [23–25]. Epigenetic modification (DNA methylation, histone modifications, microRNAs) is one the critical drivers for fetal over-nutrition and increased susceptibility to obesity development in adult life [25, 26]. Progeny born to mothers who ate an HF diet also tend to eat an HF diet when growing and have been found to develop metabolic problems [27, 28]. Thus, the generational transmission of obesity is cyclical [29].

Current treatment for metabolic syndrome relies on the long-term use of drugs in adults. However, these drugs are not always effective for all individuals and may cause certain side effects. It is important to develop a strategy to treat and prevent the occurrence of metabolic syndrome related to prenatal or postnatal HF diet in the modern world. Reprogramming refers to reversing the development of programming errors and resuming normal development through programming manipulation [30, 31]. Supplements present in the diet of pregnant women, such as vitamin D, glycine, folic acid, and fish oil, tend to attenuate the adverse consequences for progeny [32]. Resveratrol (3,4′,5-trans-trihydroxystilbene), a natural polyphenolic compound produced in numerous plant species (grapes, peanuts, cocoa, certain berries) and red wine, is an activator of sirtuin 1 (SIRT1). It makes a substantial contribution for lipid and glucose regulation by deacetylating the crucial metabolic signals relevant to the AMP-activated protein kinase-SIRT1-PPARG coactivator-1α axis [33, 34]. Resveratrol treatment has been shown to improve glucose homeostasis, lipid parameters, mitochondrial function, and body weight in rats consuming an HF diet [35]. Previous study also showed that resveratrol treatment for progeny improves some of the altered metabolic symptoms, peripheral leptin resistance, and related dysbiosis of the gut programmed by combined prenatal and postnatal HF diet exposure [36, 37]. Since maternal obesity can exert a negative effect on progeny beginning from embryo development, the potential implication of maternal resveratrol administration in reprogramming is worthy of investigating [38]. In this study, the preventive effects of maternal resveratrol supplements and its mechanisms in the male progeny of dams with high-fat/high-sucrose (HFHS) diets were investigated.

## Materials and methods

### Animals and experimental protocol

This study was carried out according to the Guide for the Care and Use of Laboratory Animals by the National Institutes of Health and approved by the Institutional Animal Care and Use Committee of Kaohsiung Chang Gung Memorial Hospital (No. 2017032704) in compliance with the principles of the 3Rs (Replacement, Reduction and Refinement). Virgin Sprague-Dawley (SD) rats (BioLASCO Taiwan Co., Ltd., Taipei, Taiwan) were placed and maintained in a facility that approved by the Association for Assessment and Accreditation of Laboratory Animal Care International of Kaohsiung Chang Gung Memorial Hospital [39]. Female rats received either a regular rat diet (Fwusow Taiwan Co., Ltd., Taichung, Taiwan; 52 g% carbohydrates, 23.5 g% protein, 4.5 g% fat, 10 g% ash, and 8 g% fiber) or an HFHS diet (D12331, Research Diets, Inc., New Brunswick, NJ, USA; 35.8 g% fat plus high sucrose [35.5 g% carbohydrate], and protein 23.0 g%) ad libitum for 8 weeks before mating and during gestation and lactation. Male SD rats were kept with female rats until mating was confirmed [36, 40]. The size of the litter was normalized after delivery immediately to conform the amount of milk and maternal care equalized. A maximum of three male progenies was taken from each dam to avoid litter effects. Male progeny were supplemented with either regular food or an HFHS diet since weaning to four-month-old and were placed in four experimental groups based on the diet regimen: CC (maternal chow diet/postnatal chow diet), CH (maternal chow diet/postnatal HFHS diet), HC (maternal HFHS diet/postnatal chow diet), and HH (maternal HFHS diet/postnatal HFHS diet) (*n* = 10–12 for each group). Additionally, a maternal intervention group (HRH) with 50 mg/L resveratrol (Sigma–Aldrich, St. Louis, MO, USA) in drinking water for dams on the maternal HFHS diet/postnatal HFHS diet during pregnancy and lactation was created for comparison. The protocol for resveratrol preparation was as follows: 50 mg of resveratrol was dissolved in 30 ml 20% 2-Hydroxypropyl-β-cyclodextrin solution (Sigma–Aldrich, St. Louis, MO, USA), then dispensed into 1 L by distilled water.

### Experimental processes for sample collections

Progeny body weight (BW) was determined every month since birth to four-month-old. At four-month-old, the rat progeny was sacrificed under anesthesia of Zoletil (Zoletil® 50; Virbac corporation, Carros, France) and xylazine (Rompun®, Bayer, Leverkusen, Germany), and blood samples were obtained by cardiac puncture as previously reported [39–41]. Retroperitoneal, mesenteric, epididymal, and subcutaneous fats were collected to measure adipose tissue weight [41]. Retroperitoneal fat was selected for further biomolecular studies for its more correlation to metabolic profiles than other fat tissues [41, 42].

### Determination of plasma parameters and histological examinations

Plasma liver enzymes and cholesterol were measured by using chemistry analyzer (FUJI DRI-CHEM NX500, Tokyo, Japan). Plasma leptin and soluble leptin receptor (sOB-R) levels were determined by ELISA (Biovendor, Brno, Czech Republic, and BlueGene, Shanghai, China). The fat tissues were fixed in 10% formalin (Wako Junyaku, Osaka, Japan), and four micrometer-thick sections were stained with hematoxylin and eosin for morphometric evaluation. Images were taken by a mounted digital camera under a Nikon Eclipse E600 microscope on 10 low-power (magnification, × 40) fields (Nikon, Melville, NY, USA).

### Quantitative reverse transcription-polymerase chain reaction (RT-qPCR)

Total RNA extracted from adipose tissue was used to generate cDNA with Moloney-murine leukemia virus reverse transcriptase as previous reported [39]. RT-qPCR was carried out for *FAS* (fatty acid synthase), *LPL* (lipoprotein lipase), leptin, and leptin receptor. *GAPDH* (glyceraldehyde 3-phosphate dehydrogenase) gene expressions were utilized to normalized the genes. qPCR was carried out with SYBR Green PCR Master Mix (Thermo Fisher Scientific, San Jose, CA) containing 10 mM forward and reverse primers. The cycling protocol was conducted as previously reported [36, 41]. The threshold cycles (Ct) were determined with Light Cycler software (ver. 1.5.0) and the relative quantification of mRNA expression was evaluated by compared Ct [39–41]. The primer sequences used were provided in Supplementary Table 1.

### Western blotting

Adipose tissue (50 mg) was homogenized and extracted with a protein extraction solution (iNtRON Biotechnology Inc., Seongnam, South Korea). After the concentration was evaluated by using protein assay kit (Bio-Rad, Hercules, CA, USA), samples were mixed with a sample buffer, boiled, and indicated to electrophoresis using sodium dodecyl sulfate-polyacrylamide gels [36]. After transfer to a polyvinylidene fluoride membrane (Millipore, Bedford, MA, USA) and blocking with phosphate-buffered saline-Tween (5% skim milk), the membranes were immersed with the following first antibodies: SIRT-1, (#ab110304, Abcam, Cambridge, MA, USA), FAS (Abcam, Cambridge, MA, USA), and LPL (Abcam, Cambridge, MA, USA) for 2 h. The membranes were then immersed for 1 h with peroxidase-labeled secondary antibody diluted in TBS-Tween after washing with 0.1% T-TBS. After soaking with TBS-Tween, the membranes were developed using the ChemiDoc XRS (Bio-Rad Laboratories, Hercules CA) to image the blots and Image Lab v5.0 (Bio-Rad, Hercules, CA, USA) to determine the density for each band as the integrated optical density after subtraction of background. The integrated optical density was factored for Ponceau red staining to correct protein loading. The relative abundances of proteins were determined with western blotting, as previously reported [41].

### Statistical analysis

Two-way analysis of variance (ANOVA) was utilized to estimate the consequence of maternal HFHS diet and postnatal HFHS diet as dependent variables. Bonferroni correction was used to determine the subsequent simple-effects. The differences between the HH and HRH groups was used by Mann-Whitney *U* test. Data were demonstrated as mean ± standard error (SEM). For all tests, two-sided *P*-values less than 0.05 were tested as arrive statistical significance by using SPSS 22.0 for Windows XP (SPSS, Inc., Chicago, IL, USA).

## Results

### Maternal resveratrol therapy lessens weight and adiposity of progeny caused by prenatal and postnatal HF diet exposure

**Fig. 1:**
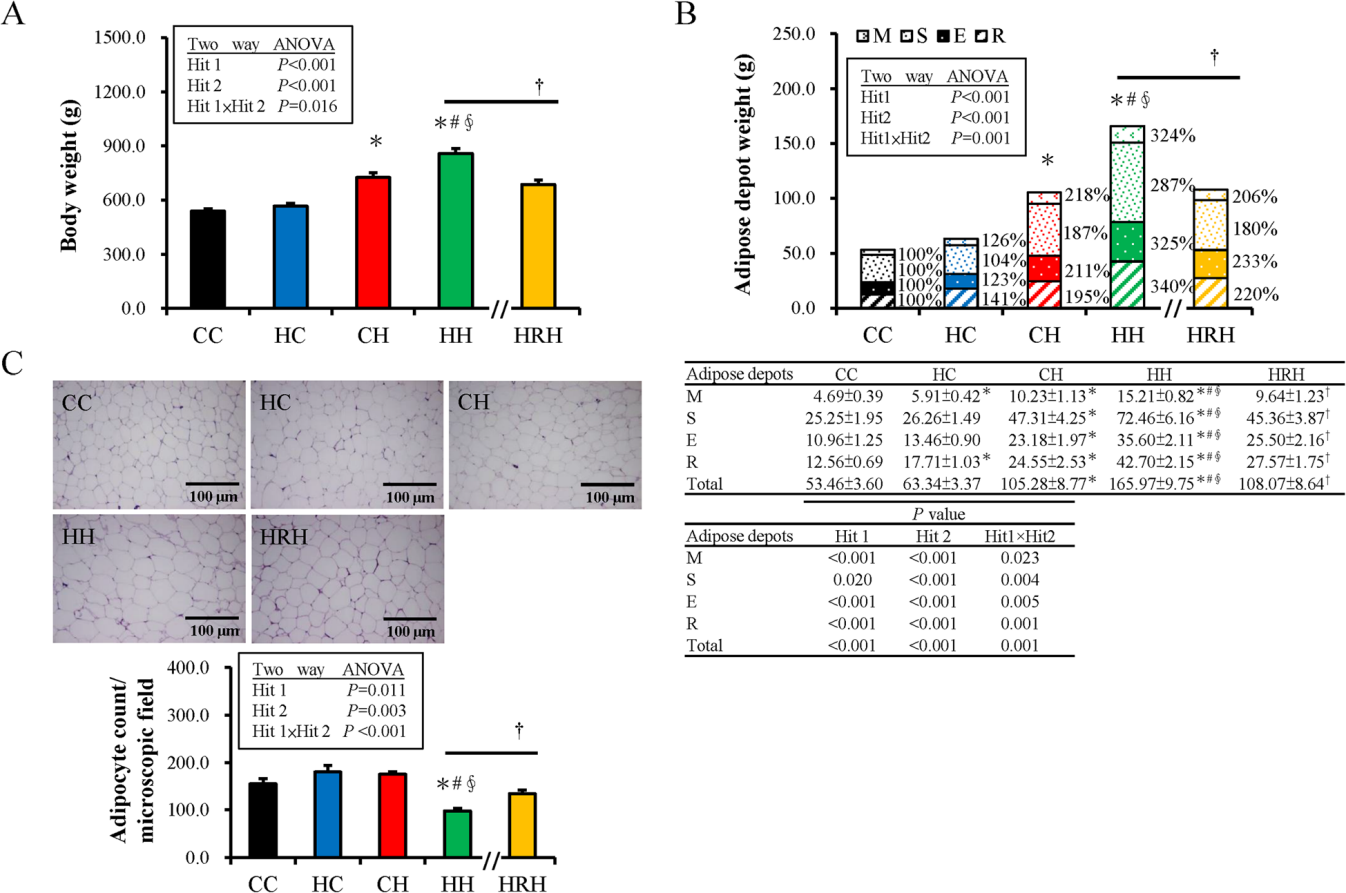
Body weight (BW) and adiposity of male progeny at four-months-old. (**a**) The BW and (**b**) total and individual adipose tissue depots weight relative the control group. Mesenteric (M), retroperitoneal (R), epididymal (E), and subcutaneous (S) fats were collected to measure adipose tissue weight; (**c**) The average adipocyte counts per microscopic field. The individual effects of maternal high-fat/high-sucrose (HFHS) diet (Hit 1) and postnatal HFHS diet (Hit 2) and their interaction (Hit 1 × Hit 2) were estimated with a two-way ANOVA. (Abbreviations: CC, maternal/postnatal control diet; HC, maternal HFHS/postnatal control diet; CH, maternal control/postnatal HFHS diet; HH, maternal/postnatal HFHS diet; and HRH, maternal HFHS diet plus maternal resveratrol/postnatal HFHS diet; *compared with CC, *P <* 0.05; #compared with HC, *P <* 0.05; §compared with CH, *P <* 0.05 by a Mann-Whitney *U* test). The Mann-Whitney *U* test was used to evaluate the therapeutic effect of resveratrol; †*P <* 0.05. (*n* = 10–14)

Supplementary Table 2 showed the body weight (BW) changes of dams in different groups. A HFHS diet led to heavier BW than control diet for dams. The birth BW and growth BW of progeny in different groups were shown in Supplementary Table 3 and Supplementary Table 4, respectively. There was a significant positive interaction between maternal HFHS (equivalent to Hit 1) and postnatal HFHS diets (Hit 2) on progeny BW and adipose tissue (Fig. 1a and b). Combined maternal HFHS and postnatal HFHS diets showed a synergetic effect leading to a higher BW and total adipose tissue weight observed in the HH group at four-months-old. The effects of the prenatal HFHS diet [BW, F (1,53) =13.92, *P* < 0.001; total adipose tissue weight, F (1, 53)  = 23.43, *P*  <  0.001], and postnatal HFHS diet [BW, F (1,53) =124.61, *P* < 0.001; total adipose tissue weight, F (1, 53)  =  112.22, *P* < 0.001] were shown respectively. Maternal and postnatal HFHS diet revealed a positive interactive effect on BW [F (1,53) =6.14, *P* = 0.016]). Furthermore, maternal and postnatal HFHS diets also resulted in the heaviest BW and total adipose tissue weight for the HH group. Maternal resveratrol treatment (HRH group) significantly decreased the BW (685.86 ± 23.95 vs. 858.79 ± 27.56 g, *P* < 0.001) and total adipose tissue weight gains (108.07 ± 8.64 vs. 165.97 ± 9.75 g, *P* < 0.001) observed in the HH group (Fig. 1a and b). For different fat depots, a two-way ANOVA analysis showed that both maternal HFHS and postnatal HFHS diets had a powerful impact on weight increase in four fat depots with Hit 1/Hit 2 interactions (Fig. 1b). In addition, a maternal HFHS diet had more effects on weight gain in visceral fat depots (retroperitoneal and mesenteric) compared to subcutaneous fat. Maternal resveratrol intervention rescued the adiposity of all four fat depots introduced by prenatal and postnatal HFHS diets. Hematoxylin and eosin staining for progeny retroperitoneal fat tissue (Fig. 1c) revealed the HH group had the least adipocyte counts (largest adipocyte size) in a fixed microscopic field than the other groups. Maternal resveratrol therapy also decreased the adipocyte size of retroperitoneal tissue caused by prenatal and postnatal HFHS diets. These results suggest a synergic effect of maternal and postnatal HFHS diets on BW and total adipose tissue weight of progeny. This effect was suppressed and reprogrammed by maternal resveratrol therapy.

**Fig. 2:**
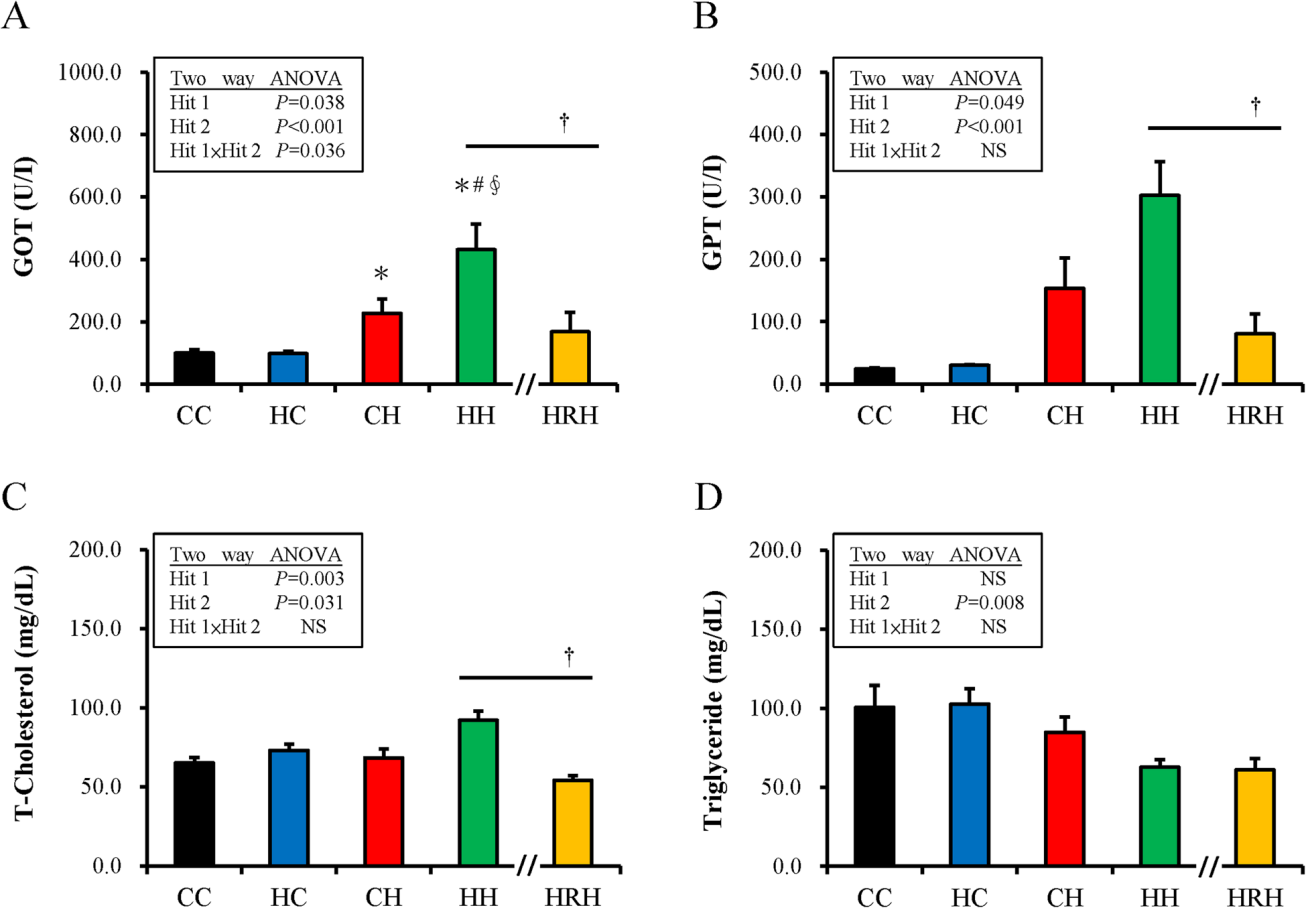
The liver function markers, total-cholesterol, and triglyceride level change with maternal/postnatal high-fat/high-sucrose (HFHS) diet and maternal resveratrol treatment. (**a**) Glutamic oxaloacetic transaminase, (**b**) glutamic pyruvic transaminase, (**c**) total cholesterol level, and (**d**) triglyceride level. The statistical significance of differences among groups was determined by a two-way ANOVA, and the Mann-Whitney *U* test was used to evaluate the therapeutic effect of resveratrol (†*P* < 0.05)

For liver function markers, glutamic oxaloacetic transaminase and glutamic pyruvic transaminase of the different groups showed that both maternal HFHS and postnatal HFHS diets significantly affected liver function (Fig. 2a and b). Resveratrol treatment (HRH) significantly improved the liver function observed in the HH group. Further, the total cholesterol level increased with both maternal and postnatal HFHS diets (Fig. 2c). Resveratrol treatment (HRH) ameliorates the increased total cholesterol level in the HH group. However, no significant difference was shown in the triglyceride level between the HH and HRH groups (Fig. 2d).

### Maternal resveratrol treatment rescued the obesity programmed by prenatal and postnatal HFHS diets by total food intake reduction and metabolic modulation

**Fig. 3:**
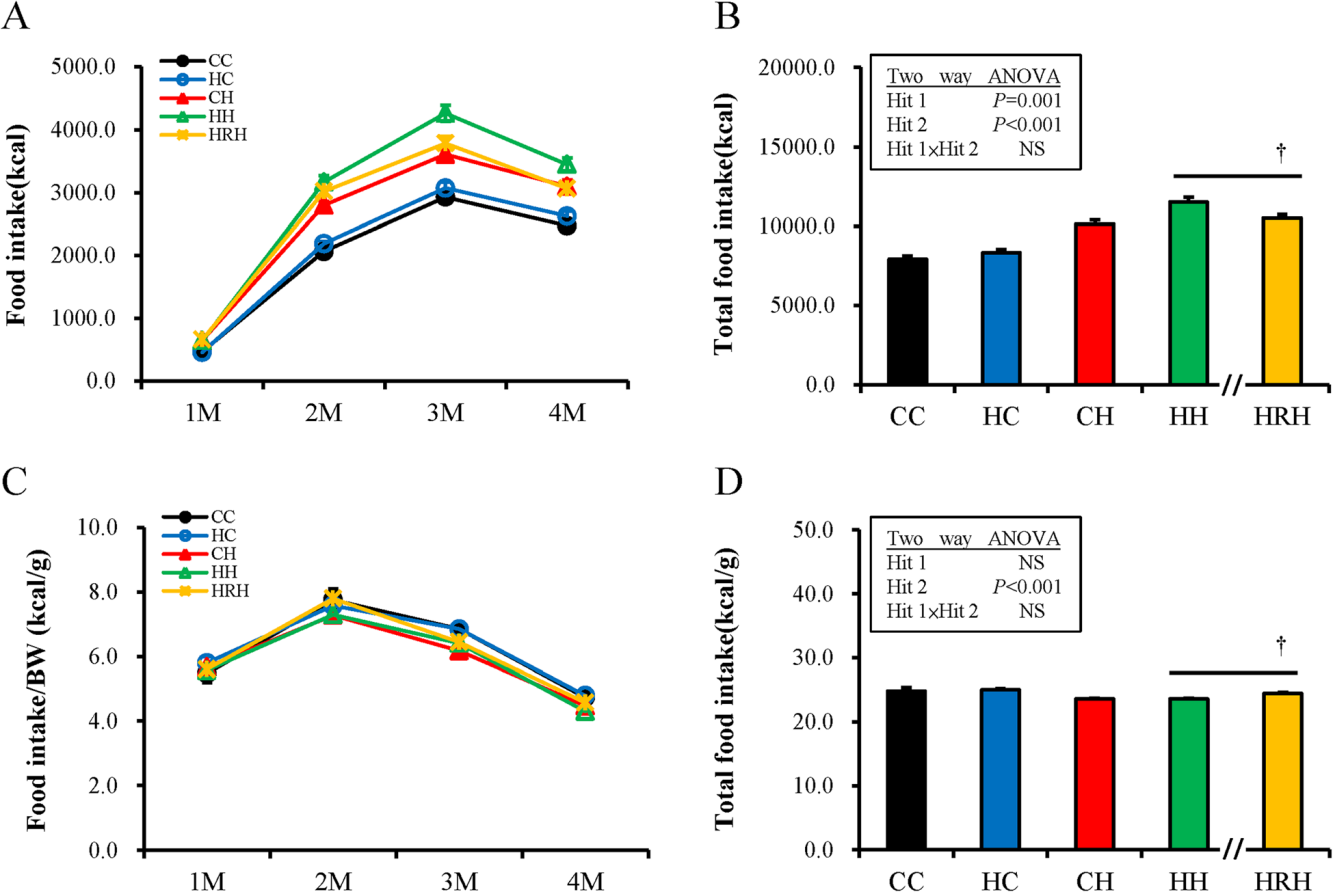
Total calorie taking and calorie taking for each unit of body weight among different groups. (**a**) Monthly calorie taking from birth to four-months-old; (**b**) Total calorie intake for the four-months period; (**c**) Monthly calorie intake per unit body weight from birth to four-months-old; (**d**) Total calorie intake per unit of body weight for the four-months period. Abbreviations: CC, maternal/postnatal control diet; HC, prenatal high-fat/high-sucrose (HFHS) /postnatal control diet; CH, prenatal control/postnatal HFHS diet; HH, maternal/postnatal HFHS diet; and HRH, maternal HFHS diet plus prenatal resveratrol/postnatal HFHS diet). The effects of maternal HFHS, postnatal HFHS diet, and their interaction were determined using two-way ANOVA. The Mann-Whitney *U* test was used to evaluate the therapeutic effect of resveratrol (†*P <* 0.05)

The monthly sum of total calorie intake is shown in Fig. 3a. For the total calorie intake over 4 months, a two-way ANOVA analysis showed that both maternal HFHS [Hit1 F (1,53) =0.11, *P* = 0.001] and postnatal HFHS diets [F (1,53) =19.62, *P* < 0.001] have significant impacts on total calorie intake without interaction [F (1, 53)  =  0.13, *P* = 0.076] (Fig. 3b).

For the total calorie intake over 4 months, the HRH group revealed less calorie intake than the HH group (10,526.41 ± 237.12 vs. 11,519.01 ± 333.50 Kcal, *P* = 0.01). On the other hand, as the calorie taking for each unit of BW was considered, progeny showed the most calorie intake per unit BW at two-months-old compared to other stages on average (Fig. 3c). For the calorie intake of per unit BW, a two-way ANOVA between groups analysis showed a significant main influence for postnatal HFHS from two- to four-month-olds without a maternal HFHS /postnatal HFHS diet interaction (Fig. 3d). It is interesting to note that the HRH group had more calorie ingestion per unit BW than the HH group for the total four-month period (24.41 ± 0.17 vs. 23.57 ± 0.09 Kcal/g, *P* < 0.001), although the total calorie intake was less than the HH group during the four-month period (HRH vs. HH: 24.41 ± 0.17 vs. 23.57 ± 0.09 Kcal/g, *P* < 0.001) (Fig. 3d). The impact of intrauterine resveratrol administration on energy expenditure seems attenuated when littermates are high-fat fed. Thus, the re-programming consequence of prenatal resveratrol on BW and adiposity of progeny occurred through both appetite reduction and metabolic modulation.

### Maternal resveratrol treatment improves the decreased SIRT1 abundance of retroperitoneal tissue mediated by the united influence of prenatal and postnatal HFHS diets

**Fig. 4:**
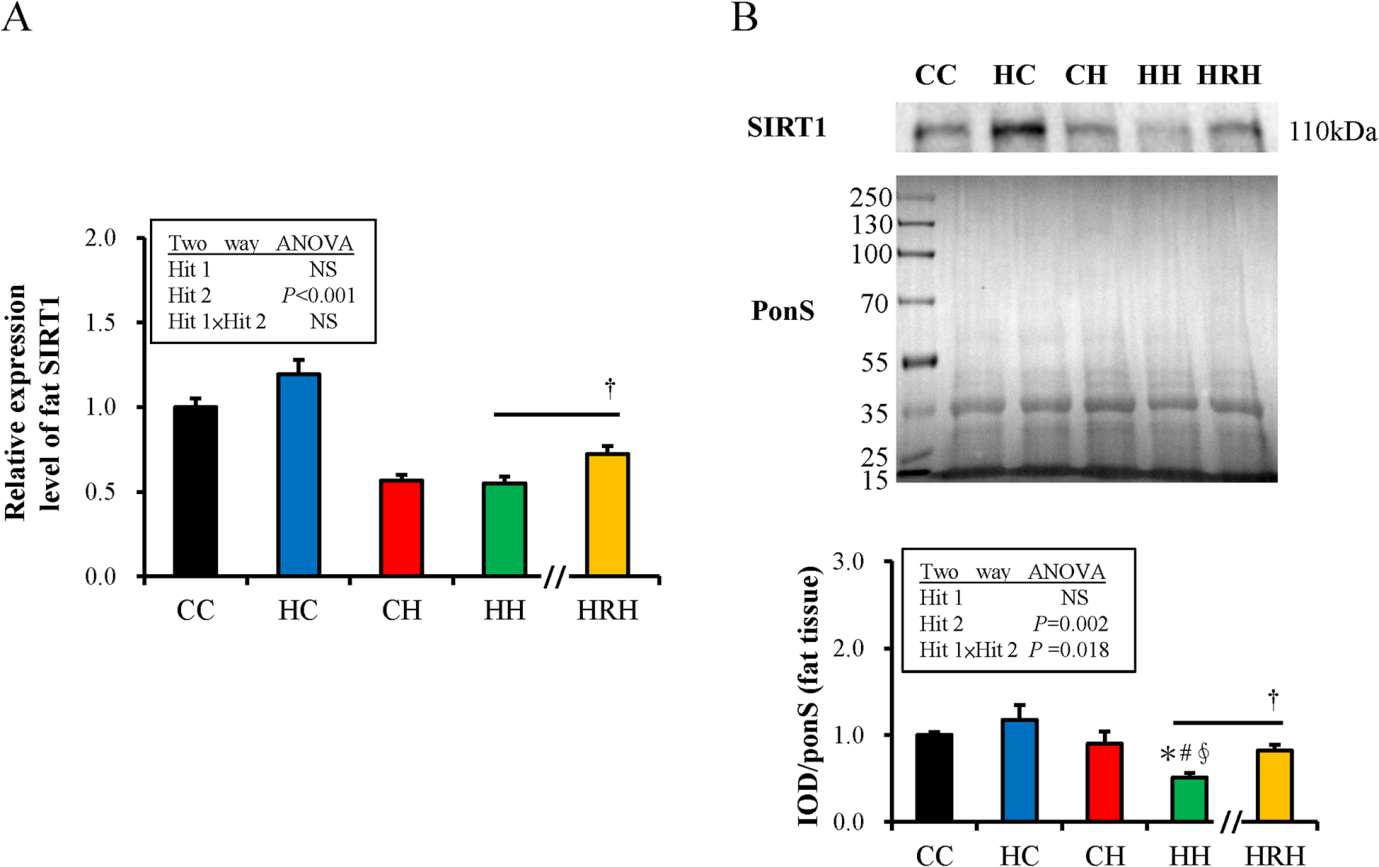
Gene expression and protein abundance of Sirtuin 1 (SIRT1) in retroperitoneal adipose tissue. **a** The relative gene expression of SIRT1. **b** The protein abundance of SIRT1. Illustrative immunoblotting and optical density quantification of SIRT-1 are shown. The relative protein amount was presented as an integrated optical density (IOD)/Ponceau S staining (Pon S). The statistical differences among groups was determined by Two-way ANOVA. Values are mean ± standard error of the mean (SEM) (*n* = 10–14 for qPCR and *n* = 8 for western blotting); *compared with CC, *p <* 0.05; #compared with HC, *P <* 0.05; §compared with CH, *P <* 0.05 by a Mann-Whitney *U* test. The effect of postnatal resveratrol treatment was evaluated by a Mann-Whitney *U* test; †*P <* 0.05. (Abbreviations: CC, maternal/postnatal control diet; HC, maternal high-fat/high-sucrose (HFHS)/postnatal control diet; CH, maternal control/postnatal HFHS diet; HH, maternal/postnatal HFHS diet)

SIRT1 makes a substantial contribution in lipid and glucose regulation by deacetylating the master metabolic and inflammatory signals. Resveratrol is known as a SIRT1 activator. Further study was conducted to investigate the manifestation of SIRT1 by an HF diet and maternal resveratrol intervention. As showed in Fig. 4a, postnatal HFHS rather than maternal HFHS exposure significantly decreased the expression of SIRT1 mRNA [Hit 1: F (1,44) =1.37, *P* = 0.248; Hit 2: F (1,44) =109.78, *P* < 0.001; H1 and H2 interaction: F (1,44) =2.573, *P* = 0.116]. Maternal resveratrol treatment (HRH group) significantly ameliorated the decrease of SIRT1 mRNA caused by maternal HFHS diet plus postnatal HFHS diet (HH group) (*P* = 0.015). Further, the abundance of SIRT1 in adipose tissue was determined by western blotting (Fig. 4b). SIRT1 abundance was also significantly attenuated in the HH group. A maternal HF diet could aggravate the decrease of SIRT1 abundance induced by a postnatal HFHS diet [Hit 1: F (1,28) =0.92, *P* = 0.346; Hit 2: F (1,28) =11.41, *P* = 0.002; H1 and H2 interaction: F (1,28) =6.27, *P* = 0.018]. It was found that maternal resveratrol treatment also improves the reduced SIRT1 protein amount in retroperitoneal adipose depot mediated by prenatal HFHS and postnatal HFHS diets (*P* = 0.005).

### Maternal resveratrol treatment attenuates leptin resistance programmed by maternal and postnatal HFHS diets

**Fig. 5:**
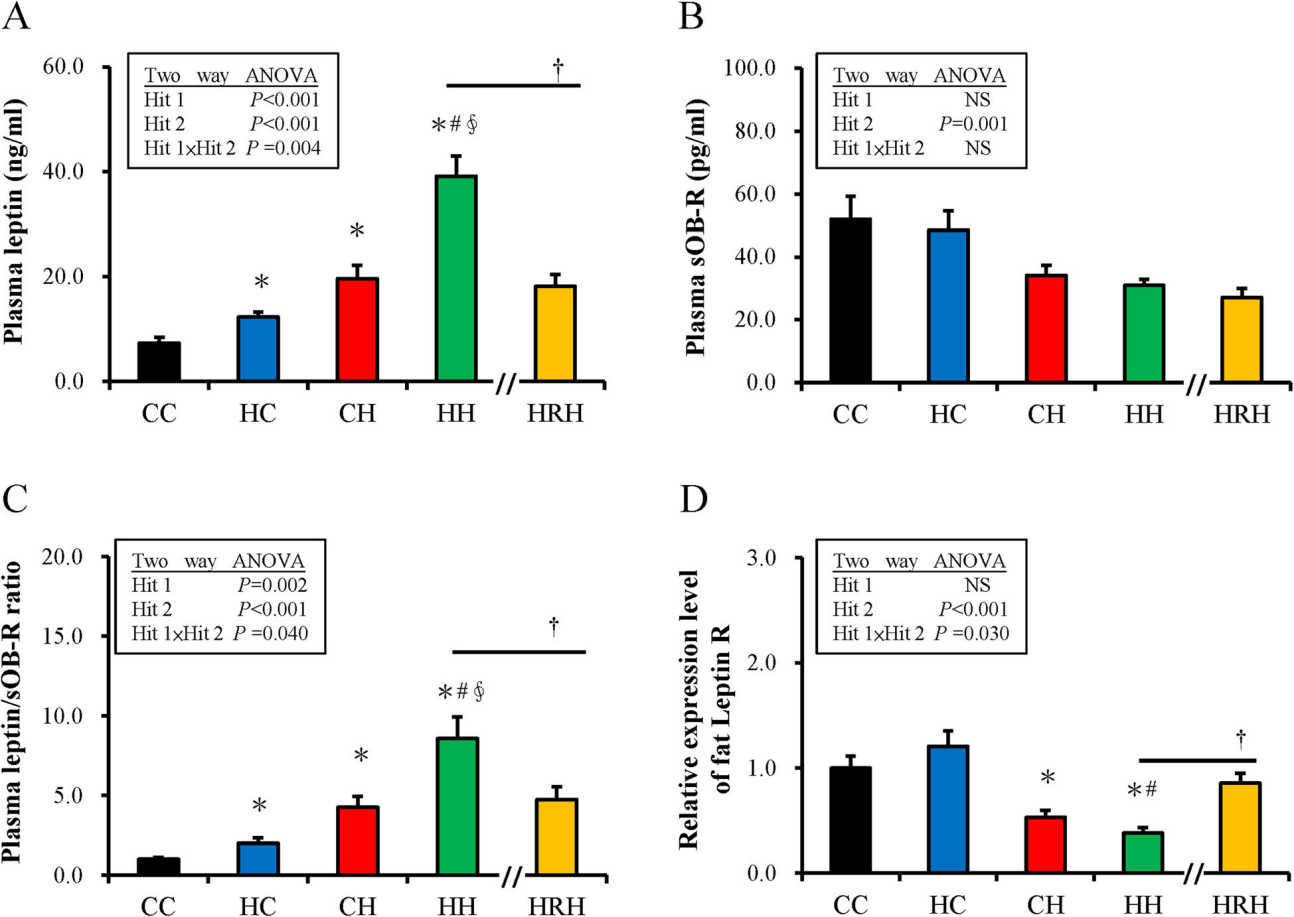
Change in Leptin related molecules induced by maternal high-fat/high-sucrose (HFHS)/postnatal HFHS exposure and maternal resveratrol treatment. **a** The level of plasma leptin. **b** and soluble leptin receptor (sOB-R). **c** The fraction of plasma leptin and sOB-R level of progeny. **d** The relative expression of leptin receptor (OB-R) gene in the retroperitoneal adipose depot. (†*P <* 0.05)

In further analysis, the plasma leptin and sOB-R levels were determined. Both maternal HFHS and postnatal HFHS diet exposure significantly increased plasma leptin levels [Hit 1: F (1,44) =25.93, *P* < 0.001; Hit2: F (1,44) =66.69, *P* < 0.001]. An interactive result of Hit 1 and Hit 2 for plasma leptin was identified [F (1,44) =9.20, *P* = 0.004]. A maternal HFHS diet acting in synergy with a postnatal HFHS diet raised plasma leptin concentration, as observed in the HH group, that could be relieved by maternal resveratrol treatment (HH vs. HRH: 39.16 ± 3.81 vs. HRH: 18.12 ± 2.26 ng/ml, *P* < 0.001) (Fig. 5a). Figure 5b showed that plasma sOB-R significantly decreased with a postnatal HFHS diet [F (1,44) =11.71, *P* = 0.001] but not prenatal HF diet exposure [F (1,44) =0.41, *P* = 0.528] without Hit 1 / Hit 2 interaction. Maternal resveratrol treatment could not restore the decrease of plasma sOB-R in the HH group (*P* = 0.356). The Ratio of total leptin to sOB-R level is a biological marker for leptin resistance [43–45]. Thus, the ratio of leptin to sOB-R was examined and both prenatal HFHS and postnatal HFHS diets increased the leptin/sOB-R ratio in progeny [Hit 1: F (1,44) = 0.38, *P* = 0.002; Hit 2: F (1,44) = 59.54, *P* < 0.001; H1 and H2 interaction: F (1,44) = 5.02, *P* = 0.040]. Progeny that received prenatal HFHS and postnatal HFHS diets had the highest leptin/sOB-R ratio. Maternal resveratrol treatment ameliorated the high leptin/sOB-R ratio induced by maternal HFHS and postnatal HFHS diet exposure (HH vs. HRH: 8.57 ± 1.35 vs. 4.73 ± 0.82, *P* = 0.049) (Fig. 5c). Thereafter, the gene expressions of the leptin receptor (OB-R) in retroperitoneal adipose tissue were evaluated using qPCR. Similar to sOB-R level, a postnatal HFHS diet, but not prenatal HFHS diet, attenuate the expression of OB-R mRNA in the retroperitoneal adipose depot of progeny (Fig. 5d). Prescription of maternal resveratrol partially rescued the decreased gene expression of OB-R induced by prenatal and postnatal HFHS diet exposure (*P* = 0.001).

### Maternal resveratrol therapy alleviates lipogenesis and increases lipolysis to achieve an anti-obesity effect

**Fig. 6:**
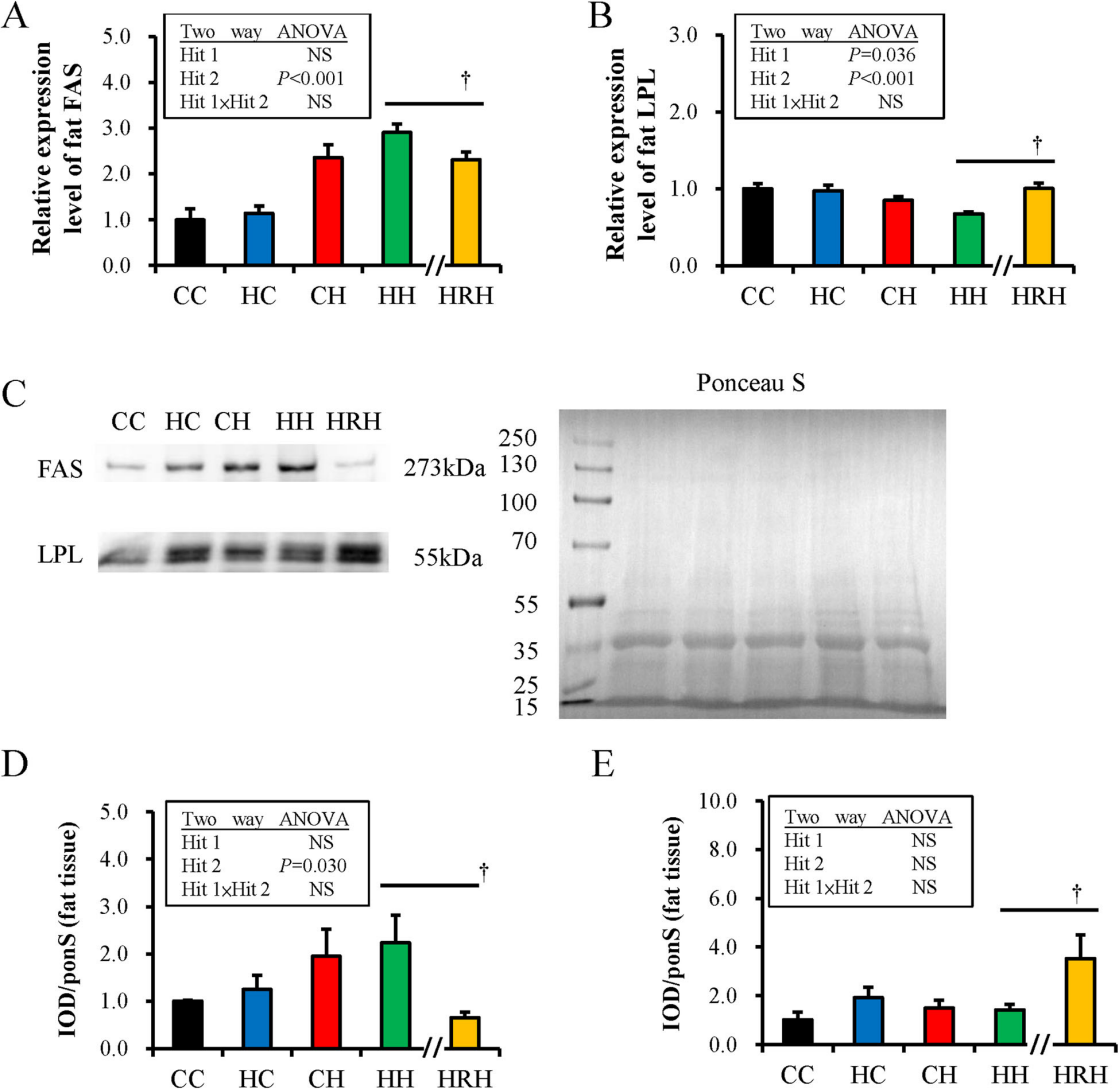
Target gene expression and protein levels in rat retroperitoneal depot of progeny. The mRNA expressions of (**a**) free fatty acid synthase (*FAS*) and (**b**) lipoprotein lipase (*LPL*) were determined by RT-PCR. The protein abundance was normalized with Ponceau S staining (ponS). (**c**) Representative band densities are illustrated. Relative abundance of (**d**) FAS and (**e**) LPL were quantified. The value is expressed as a multiple relative to CC, maternal/postnatal control diet. Protein amount was shown as IOD/ponS. (†*P <* 0.05)

Maternal resveratrol supplementation seems to result in an anti-obesity effect on progeny partially through metabolic modulation. Thus, the change in the manifestation of *FAS* and *LPL* genes in the retroperitoneal adipose depot was investigated to determine the lipid modulatory effects of resveratrol. Figures 6a and b showed that postnatal HFHS diet exposure significantly amplified the expression of the *FAS* gene [F (1,36) =40.63, *P* < 0.001] in retroperitoneal fat tissue, while both maternal HFHS [F (1,44) =4.67, *P* = 0.036] and postnatal HFHS diets [F (1,44) =20.22, *P* < 0.001] attenuated the expression of the *LPL* gene in adipose tissue without a Hit 1/Hit 2 interaction. The relative protein abundances of FAS and LPL in retroperitoneal adipose tissue were also evaluated by western blotting. Figure 6c showed that the abundance of FAS increased with postnatal HFHS diet exposure [F (1,31) =5.14, *P* = 0.030] without Hit 1/Hit 2 interaction. The abundance of LPL was not influenced by maternal or postnatal HFHS diet (Fig. 6d). Resveratrol treatment (HRH) significantly decreased the gene expression and protein abundance of FAS of the retroperitoneal adipose depot as compared with the HH group (Fig. 6a and c). Resveratrol treatment (HRH) also increased the gene expression and protein abundance of LPL observed in the HH group (Fig. 6b and d). Therefore, resveratrol decreases lipogenesis and increases lipolysis, resulting in an anti-obesity effect.

## Discussion

This study showed that maternal resveratrol treatment lessens the weight and adiposity of progeny that were programmed by combined prenatal and postnatal HF diets. Maternal resveratrol supplementation achieved a therapeutic effect for progeny through food intake reduction and metabolic modulation. Maternal resveratrol therapy also ameliorated the decreased SIRT1 protein abundance of retroperitoneal tissue mediated by the synergetic influence of maternal and postnatal HFHS diet exposure. Moreover, maternal resveratrol treatment could alleviate the altered leptin/sOB-R ratio of progeny that had been programmed by prenatal and postnatal HFHS diets. Maternal resveratrol therapy decreased lipogenesis and increased lipolysis for progeny, resulting in an anti-obesity effect. Thus, prenatal resveratrol has the potential to serve as a preventative strategy for HFHS diet-induced obesity.

A previous study showed that resveratrol treatment for progeny after weaning could lessen the adiposity programmed by combined maternal plus postnatal HFHS diets through lessening intake or inducing metabolic changes [36]. Here, resveratrol was administrated for dams rather than progeny to determine the preventative effect of maternal resveratrol treatment for prenatal and postnatal HFHS diet exposure. The study showed that maternal resveratrol supplementation also has an anti-obesity/anti-adiposity effect for progeny that were exposed to maternal and postnatal HFHS diets through lessening intake or inducing metabolic changes. Similar to postnatal resveratrol treatment [36], maternal resveratrol therapy could also activate the decreased SIRT1 amount in progeny retroperitoneal fat depot programmed by prenatal and postnatal HFHS diets. Both prenatal and postnatal resveratrol supplementation could decrease the altered plasma leptin levels in progeny induced by maternal and postnatal HFHS diet exposure. Thus, maternal resveratrol treatment has reprogramming effects on the adverse impacts of prenatal and postnatal HFHS diet environments. However, although both maternal resveratrol and postnatal resveratrol treatments demonstrated anti-obesity and metabolic modulatory effects for prenatal and postnatal HFHS diets, they utilized several different therapeutic mechanisms. First, in this study, when the calorie intake per unit BW was considered, the HRH group had more calorie ingestion per unit BW than the HH group, although the total calorie intake was less than the HH group during the four-month period. Thus, the reprogramming effects of maternal resveratrol treatment on the adiposity of progeny mainly occur through metabolic modulation. Second, postnatal resveratrol treatment exceedingly increased the gene expression of leptin receptors in retroperitoneal adipose tissue of progeny (almost 40 times compared to the control group). In contrast, that maternal resveratrol treatment (HRH group) could only partially ameliorate the decreased expression of the leptin receptor mRNA observed in the HH group and does not lead to as much improvement as postnatal resveratrol therapy. Thus, the DNA methylation of leptin receptors was not analyzed in this study. Third, maternal resveratrol treatment significantly ameliorated the decrease in expression of the *LPL* gene in the HH group, while postnatal resveratrol treatment does not influence the expression of the *LPL* gene.

A study from Hsu, et al. had shown that maternal HFHS diet reduce adiponectin, SIRT1, p-AKT, and brain-derived neurotrophic factor (BDNF) of fetal brain and adult progeny hippocampus [46]. Maternal resveratrol treatment could renovate hippocampal p-AKT and BDNF of adult progeny with prenatal HFHS plus postnatal HFHS diet [46]. Maternal resveratrol supplementation seems reach both the central and peripheral tissues of the progeny. Maternal HF diet was proven to increase the proliferation of hypothalamic peptide-producing neurons of progeny and was claimed related to long-term preference for HF diet and obesity [47]. Whether prenatal resveratrol can modulate the altered neurotransmitters induced by maternal HF exposure is an interesting issue and need further studies. Maternal resveratrol therapy was also reported to reverse the insulin resistance induced by combined maternal HF plus postnatal HF diet exposure [46, 48]. The beneficial effects of resveratrol in HF exposed dams may have been indirect, as possibly related to glucose-lowering and insulin-sensitizing activities.

Resveratrol has many biological effects, such as anticancer, antioxidant, and anti-inflammatory characteristics [49]. Resveratrol can also be used to prevent heart disease and other diseases associated with aging. Other studies have revealed that resveratrol can prevent hepatic lipid accumulation induced by an HFHS diet [49–51]. The influence of maternal resveratrol supplementation on progeny has also been discovered recently. It has been shown that maternal resveratrol therapy can prevent cognitive decline in senescent mice progeny [52]. Tanaka et al. recently reported that prenatal resveratrol administration during lactation could decrease liver fatty acid synthesis of adult male rat progeny [53]. Maternal resveratrol administration can induce the activation of AMP-activated protein kinase through SIRT1 upregulation in the livers of adult male rat progeny. Therefore, the proteolytic processing of sterol regulatory element-binding protein-1c suppresses the gene expression of downstream lipogenesis-related enzymes, such as FAS and acetyl-CoA carboxylase in the livers of adult male progeny [53]. It has also previously been shown that maternal resveratrol intake protects against dysfunction of the islet and gestational diabetes-induced glucose intolerance in rat progeny [54]. Resveratrol supplementation for HFHS diet-fed pregnant mice also could promote brown and beige adipocyte development, thereby increasing energy expenditure and protecting progeny from HFHS diet-induced obesity and insulin resistance in adulthood. Thus, maternal resveratrol administration seems to protect progeny against postnatal HF diet-induced obesity [55, 56]. Here, further evidence is provided that showing the anti-obesity/anti-adiposity effect of maternal resveratrol treatment on prenatal and postnatal HF diet exposure.

The multi-faceted actions of resveratrol are mediated by its regulatory functions on key transcription factors and kinases, such as NF-kB, cytochrome P450, p53, mTOR, cyclins, AKT, AMPK, SIRT1, PGC-1α that modulate metabolism [35, 57]. Whether maternal resveratrol still exerts such versatile functions for progeny is not clear now. Previous study had shown that resveratrol therapy can improve the plasma propionate level, dysregulated metabolic parameters, and dysbiosis related to maternal HFHS plus postnatal HFHS diet exposure [37]. More studies are needed to clarify the effect of maternal gut microbiota changes on the metabolism of progeny.

Caloric restriction has been shown to elongate lifespan in mammals through the activation of SIRT1 that regulate cellular energy metabolism and redox state [58]. Resveratrol seems like caloric restriction by enhancing SIRT1/PGC-1α signaling pathway and mitochondrial biogenesis that linked to the regulation of the vitagene system and metabolism homeostasis [58–61]. Given the relationship between redox status and the vitagene network and its possible biological relevance in metabolism homeostasis, the contribution of maternal resveratrol to redox status and vitagene systems need further to be explored.

Total plasma leptin/sOB-R ratio is considered a biological marker for leptin resistance [43–45]. BW decrease in obese adults is inclined to decrease the leptin/sOB-R ratio, implying an improvement in leptin resistance [62]. Both maternal HF and postnatal HF diets led to increased leptin serum levels, but only postnatal HF decreased plasma sOB-R concentrations in the progeny. Prenatal resveratrol can attenuate plasma leptin but not change plasma sOB-R levels induced by prenatal and postnatal HF diet exposure. Apart from the central leptin resistance, which develops in the nervous system, peripheral leptin resistance has been observed in adipose tissue [63]. Obese individuals revealed a lower leptin receptor gene expression in adipose tissue than lean ones [64]. Although maternal resveratrol treatment did not enhance plasma sOB-R levels in this study, it was found that maternal resveratrol therapy augmented the gene expression of leptin receptors in the retroperitoneal fat depot. Thus, maternal resveratrol treatment appears to have a beneficial effect on the peripheral leptin resistance of progeny visceral fat tissue with prenatal and postnatal HFHS diet exposure.

The tendency to lower plasma triglycerides responses to post-weaning high-fat meals in HH and HRH groups, with respect to control meals in CC and HC groups, appears paradoxical in this study. This might be explained by diet components difference. In this study, the fat component of HFHS diet was provide by coconut oil. Coconut oil has been shown to decrease the triglyceride level for normal rats and hepatosteatosis condition attributed to its medium-chain saturated fatty acids [65–67].

### Study strengths and limitations

The strength of this study is that it provide the potential and possible mechanism for maternal resveratrol in preventing the occurrence of metabolic syndrome related to prenatal or postnatal HFHS diet. This reprogramming effect for leptin resistance using prenatal resveratrol treatment is interesting, however, there are several limitations. First, to conclude metabolic regulation is difficult by solely measuring food intake and weight gain because food digestion/absorption can differ between groups, more data are needed to support it. Second, the effect of resveratrol on leptin and soluble leptin receptor may result from its beneficial effect on adiposity scores. Whether the obesity preventive effect of maternal resveratrol for progeny still exists with different diet components or different species also needs further testing. In this study, the plasma level of resveratrol and DNA methylation status of leptin receptors were not determined. The dose of resveratrol used in this study was about 10 mg/kg/day. In one report, plasma level of resveratrol was 96.5 ± 12.3 nmol/L in rats with oral taking 20 mg/kg/day for 24 h [68]. In a recent report, Zou et al., have shown that 200 mg/kg/day of resveratrol ameliorate the gestational weight gain in C57BL/6 J mice [56]. This protective effect for BW gain during pregnancy is not observed in this study (Supplementary Table 2). The dosage of resveratrol may account for this difference. The best therapeutic dosage and duration to re-program the prenatal/postnatal HF induced obesity for progeny also need to be identified.

## Conclusion

In conclusion, maternal resveratrol treatment reprograms the adiposity programmed by maternal and postnatal HFHS diets through lipid metabolic modulation. It means maternal resveratrol supplementation can prevent obesity of progeny aggravated by prenatal and postnatal HF exposure. Maternal resveratrol can enhance SIRT1 abundance, decrease *FAS* gene expression, and promote *LPL* gene expression in the retroperitoneal adipose depot programmed by combined maternal and postnatal HF diets. This study provides a new reprogramming role for the anti-obesity effect of maternal resveratrol supplements.

## Supplementary information

**Additional file 1: Supplementary Table 1.** Primer sequences used for qRT-PCR. **Supplementary Table 2.** The body weight changes of dams in different groups. **Supplementary Table 3.** The birth body weight (BW) of offspring in different groups. **Supplementary Table 4.** The body weight of offspring in different groups.

## Data Availability

The dataset supporting the conclusions of this article is available upon request for corresponding author after publication.

## Abbreviations

HF: High-fat
HFHS: High-fat/high-sucrose
SIRT1: Sirtuin 1
BW: Body weight
SD: Sprague-Dawley
FAS: Fatty acid synthase
LPL: Lipoprotein lipase
GAPDH: Glyceraldehyde 3-phosphate dehydrogenase
Ct: Threshold cycles
ANOVA: Analysis of variance
DOHaD: Developmental origins of health and disease
RT-qPCR: Quantitative reverse transcription-polymerase chain reaction
OB-R: Leptin receptor
CC: Soluble leptin receptor, maternal/postnatal control diet
HC: Maternal high-fat/postnatal control diet
CH: Maternal control/postnatal high-fat diet
HH: Maternal/postnatal high-fat diet
HRH: Maternal high-fat diet plus maternal resveratrol/postnatal high-fat diet
