# Supplementary material for: Resveratrol intake during pregnancy and lactation re-programs adiposity and ameliorates leptin resistance in male progeny induced by maternal high-fat/high sucrose plus postnatal high-fat/high sucrose diets via fat metabolism regulation
Source: Lipids Health Dis. 2020 Jul 25;19:174. doi: 10.1186/s12944-020-01349-w (PMC7382831; doi:10.1186/s12944-020-01349-w)
Supplement: Supplementary file 1 — Additional file 1: Supplementary Table 1. Primer sequences used for qRT-PCR. Supplementary Table 2. The body weight changes of dams in different groups. Supplementary Table 3. The birth body weight (BW) of offspring in different groups. Supplementary Table 4. The body weight of offspring in different groups. [file 12944_2020_1349_MOESM1_ESM.doc]

Supplementary Table 1. Primer sequences used for qRT-PCR

| FAS | Forward | 5'-AGATCCTGGAACGTGAACATGA-3' |
| --- | --- | --- |
|  | Reverse | 5'-GCCGTACTTCACGAATGGGT-3' |
| *LPL* | Forward | 5'-GTACAGTCTTGGAGCCCATGC-3' |
|  | Reverse | 5'-GCCAGTAATTCTATTGACCTTCTTGTT-3' |
| *Leptin* | Forward | 5'-CGGTTCCTGTGGCTTTGGT-3' |
|  | Reverse | 5'-CCGACTGCGTGTGTGAAATG-3' |
| *Leptin R* | Forward | 5'-ACTGGGACATAGAGTGCTGGAT-3' |
|  | Reverse | 5'-GTTGCACTGGACAGTCTGAA AG-3' |
| *SIRT1* | Forward | 5'-TGTTTCCTGTGGGATACCTGA-3' |
|  | Reverse | 5'-TGAAGAATGGTCTTGGGTCTTT-3' |
| *GAPDH* | Forward | 5'-TCTTGTGCAGTGCCAGCCTC-3' |
|  | Reverse | 5'-GTCACAAGAGAAGGCAGCCCTGG-3' |

Supplementary Table 2. The body weight changes of dams in different groups

|  | W8 | W17 | P19 |
| --- | --- | --- | --- |
| C | 213.63±2.63 | 272.20±4.93 | 345.50±22.68 |
| H | 214.33±4.91 | 310.00±8.19^*^ | 361.00±12.06 |
| HR | 213.67±6.33 | 328.97±13.90^**^ | 412.33±29.04 |
| * as compared with C, *P*<0.05；** as compared with C, *P*<0.01 | | | |


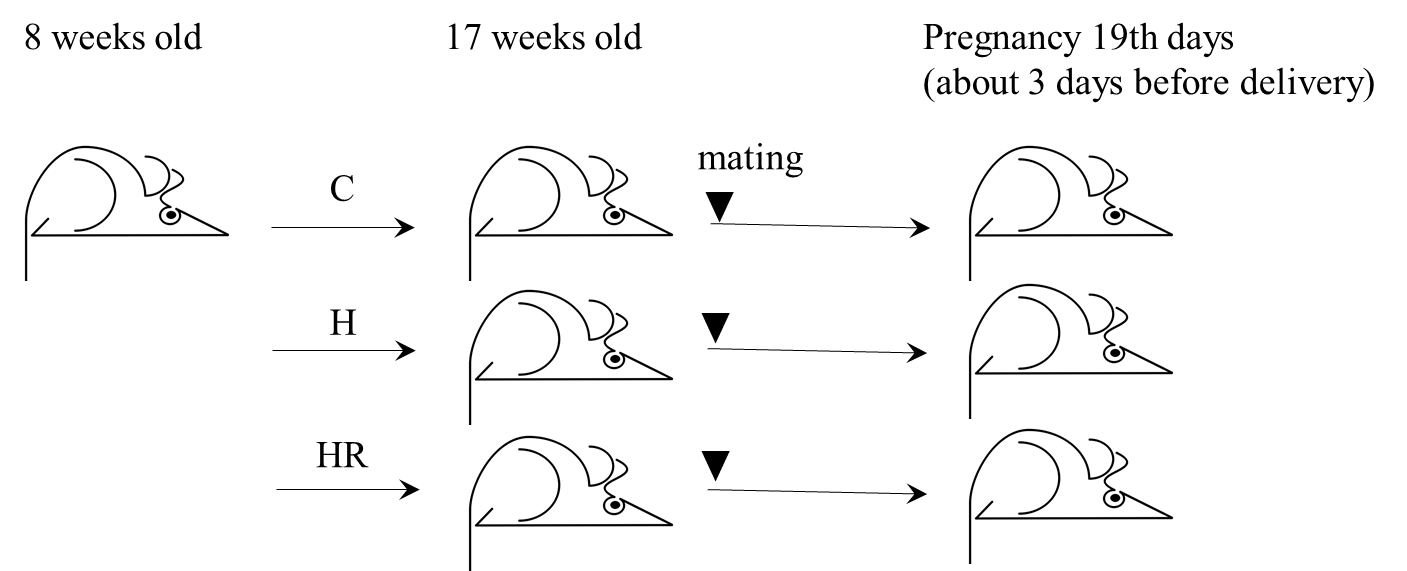


C: dam on control diet; H: dam on high-fat high-sucrose diet; HR: dam on high-fat high-sucrose diet plus resveratrol treatment; W8: eight-wee-old; W17:17-week-old, P19: pregnancy at 19^th^ days (it is about 3 days before delivery)

Supplementary Table 3. The birth body weight (BW) of offspring in different groups

|  | BW of day 2 after birth | *P*_1_ | *P*_2_ |
| --- | --- | --- | --- |
| C | 7.46±0.17 | - | - |
| H | 6.87±0.10 | 0.002 | - |
| HR | 7.92±0.10 | 0.037 | <0.001 |
| *P*_1_ as compared with C  *P*_2_ as compared with H | | | |

C: progeny with maternal control diet; H: progeny with maternal high-fat high-sucrose diet; HR: progeny with high-fat high-sucrose diet plus resveratrol treatment

Supplementary Table 4. The body weight of offspring in different groups

|  | | | | | |  | *P* value | | |
| --- | --- | --- | --- | --- | --- | --- | --- | --- | --- |
| Months | CC | HC | CH | HH | HRH |  | Hit 1 | Hit 2 | Hit 1× Hit 2 |
| 1M | 87.23±5.38 | 79.73±3.29 | 114.68±1.62 | 115.06±1.57 | 117.91±3.81 |  | NS | <0.001 | NS |
| 2M | 266.31±6.48 | 289.38±6.42 | 384.56±8.46 | 434.49±15.03 | 387.43±7.35^†^ |  | <0.001 | <0.001 | NS |
| 3M | 427.93±10.83 | 448.46±10.83 | 584.31±16.32 | 665.93±20.98 | 587.64±15.58^†^ |  | 0.002 | <0.001 | NS |
| 4M | 539.36±12.97 | 566.23±14.16 | 725.63±24.61^*^ | 858.79±27.56 ^#&^ | 685.86±23.95^††^ |  | <0.001 | <0.001 | 0.016 |
| ^*^ as compared with CC, *P*<0.001; ^#^ as compared with HC, *P*<0.001; ^&^as compared with CH, *P*=0.001;  ^†^ as compared with HH, *P*<0.05; ^††^as compared with HH, *P*<0.001  Abbreviations: CC, maternal/postnatal control diet; HC, maternal high-fat/high-sucrose (HFHS) /postnatal control diet; CH, maternal control/postnatal HFHS diet; HH, maternal/postnatal HFHS diet; and HRH, maternal HFHS diet plus maternal resveratrol/postnatal HFHS diet); M: month | | | | | | | | | |
